# CD20-Negative Primary Intestinal Diffuse Large B-cell Lymphoma With TP53 Mutation in Poorly Controlled Celiac Disease: A Diagnostic Challenge

**DOI:** 10.7759/cureus.109965

**Published:** 2026-05-31

**Authors:** Chaimae Bekhakh, Anass Haloui, Nassira Karich, Amal Bennani

**Affiliations:** 1 Pathology Department, Mohammed VI University Hospital, Faculty of Medicine and Pharmacy of Oujda, Mohammed First University of Oujda, Oujda, MAR

**Keywords:** cd20-negative, cd20-negative primary intestinal diffuse large b-cell lymphoma, celiac disease, intestinal lymphoma, large b-cell lymphoma, tp53 mutation

## Abstract

Celiac disease (CD) is associated with an increased risk of intestinal lymphoma, predominantly of T-cell origin. Primary intestinal large B-cell lymphoma arising in the setting of CD is exceptionally rare. We report the case of a 34-year-old woman with poorly controlled CD who presented with abdominal pain, weight loss, and diarrhea. CT revealed a large jejunal mass. The patient underwent segmental jejunal resection with lymph node dissection.

Histological examination showed a diffuse proliferation of large atypical lymphoid cells with focal anaplastic features. Immunohistochemical analysis demonstrated positivity for CD19, CD79a, PAX5, CD2, and perforin, whereas CD20, CD3, CD30, CD138, MUM1, and ALK were negative. Epstein-Barr virus-encoded RNA in situ hybridization was also negative. CD103 highlighted reactive intraepithelial lymphocytosis consistent with active CD.

Next-generation sequencing (NGS) identified a pathogenic TP53 mutation associated with p53 overexpression. PCR analysis confirmed B-cell clonality. The final diagnosis was CD20-negative primary intestinal large B-cell lymphoma with a TP53 mutation arising in the context of poorly controlled CD, a combination that, to the best of our knowledge, has not been previously reported in the literature.

This case highlights the diagnostic challenges posed by intestinal lymphomas associated with CD and emphasizes the importance of integrating histopathological, immunophenotypic, and molecular findings to achieve an accurate diagnosis and provide prognostic assessment.

## Introduction

Celiac disease (CD) is a chronic immune-mediated enteropathy affecting approximately 1% of the global population. It results from an abnormal immune response to dietary gluten in genetically predisposed individuals [1]. Beyond its gastrointestinal manifestations, CD is associated with a significantly increased risk of malignant complications, most notably intestinal lymphomas [2]. The relative risk of lymphoma in patients with CD is estimated to be two- to four-fold higher than in the general population, and this risk is further increased in cases of poor adherence to a gluten-free diet [3].

Enteropathy-associated T-cell lymphoma (EATL, formerly type I) and monomorphic epitheliotropic intestinal T-cell lymphoma (MEITL, formerly EATL type II) are the predominant lymphoma subtypes associated with CD. They arise from chronically activated intraepithelial T lymphocytes (IELs) [4]. In contrast, primary intestinal B-cell lymphomas occurring in the setting of CD are exceptionally rare, and only a limited number of cases have been reported in the literature [5]. Moreover, cases with an atypical immunophenotype, including loss of CD20 expression, are exceedingly uncommon and remain poorly characterized.

TP53 mutations represent one of the most frequent genetic alterations in aggressive B-cell lymphomas and are associated with chemotherapy resistance and adverse prognosis [6]. However, their role in primary intestinal B-cell lymphomas arising in CD, particularly in cases with aberrant immunophenotypic profiles, has not been systematically described.

Herein, we report a case of CD20-negative primary intestinal large B-cell lymphoma with TP53 mutation occurring in a 34-year-old woman with poorly controlled CD, comprehensively characterized by immunohistochemistry and next-generation sequencing (NGS). To the best of our knowledge, this combination of features has not been previously reported in the literature.

## Case presentation

A 34-year-old woman with poorly controlled CD presented with progressive abdominal pain, weight loss, and diarrhea. Workup revealed microcytic anemia, hypoalbuminemia, elevated inflammatory markers and lactate dehydrogenase, and markedly raised anti-tTG IgA levels (Table 1).

**Table 1:** Laboratory findings and a summary of biological values

Parameter	Result	Reference Range	Interpretation
Hemoglobin	9.2 g/dL (Low)	12–16 g/dL	Microcytic anemia
Mean Corpuscular Volume (MCV)	72 fL (Low)	80–100 fL	Consistent with iron deficiency/malabsorption
Albumin	28 g/L (Low)	35–50 g/L	Hypoalbuminemia related to malabsorption/inflammation
C-reactive Protein (CRP)	48 mg/L (Elevated)	<5 mg/L	Active inflammatory process
Lactate Dehydrogenase (LDH)	620 U/L (Elevated)	120–250 U/L	Suggestive of high cellular turnover/tissue injury
Anti-tissue Transglutaminase IgA (anti-tTG IgA)	>200 U/mL (Markedly elevated)	Negative/low normal	Active uncontrolled celiac disease

Contrast-enhanced abdominal CT scan demonstrated a large proximal jejunal mass measuring 235 × 47 mm with heterogeneous enhancement, associated with mesenteric lymphadenopathy, as illustrated in Figure 1.

**Figure 1:**
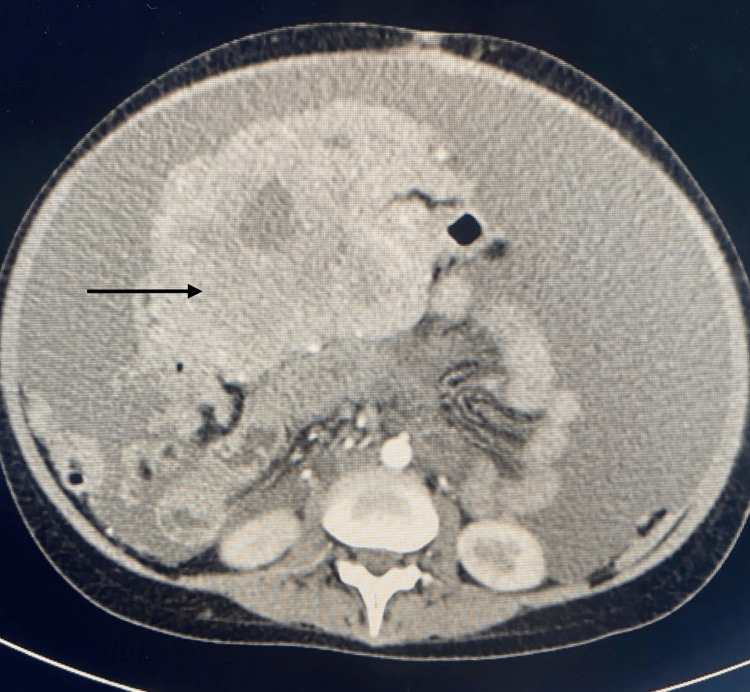
Abdominal CT scan showing a large proximal jejunal mass with heterogeneous enhancement (black arrow).

Histology showed a diffuse proliferation of large atypical lymphoid cells with frequent mitoses and focal anaplastic features, against a background of Marsh grade III mucosal changes (Figure 2).

**Figure 2:**
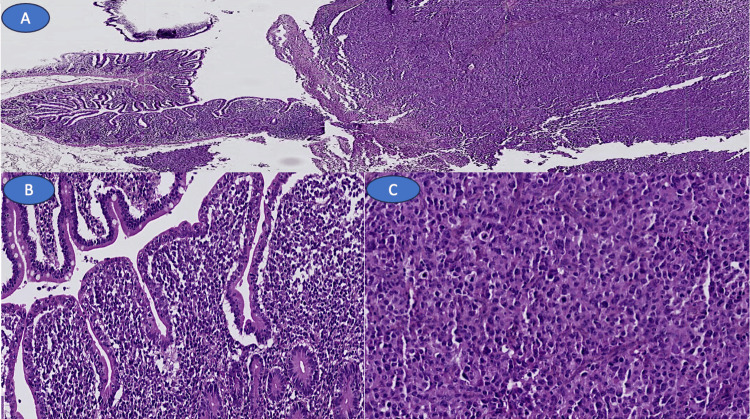
(A) ×10: Ileal mucosa extensively infiltrated by a diffuse proliferation of large lymphoid cells; (B) ×20: Villous atrophy with dense lymphocytic infiltrate consistent with celiac disease; (C) ×40: Diffuse proliferation of atypical large lymphoid cells (H&E stain).

An extensive immunohistochemical workup was then performed, and the results are summarized in Table 2 and illustrated in Figure 3.

**Table 2:** Immunohistochemical findings and phenotypic interpretation B-NHL: B-cell non-Hodgkin lymphoma; DLBCL: diffuse large B-cell lymphoma; CLL: chronic lymphocytic leukemia; SLL: small lymphocytic lymphoma; EBER: Epstein-Barr virus-encoded small RNA; IELs: intraepithelial T lymphocytes; NGS: next-generation sequencing

Marker	Result	Cell Population	Interpretation	
CD19	Positive (+)	Neoplastic cells	B-cell lineage marker; confirms B-cell origin	
CD79a	Positive (+) (heterogeneous)	Neoplastic cells	B-cell lineage marker	
PAX5	Positive (+) (clear, low intensity)	Neoplastic cells	B-cell transcription factor; key lineage determinant	
CD2	Positive (+)	Neoplastic cells	T-cell antigen; aberrant expression on aggressive B-cell lymphoma	
Perforin	Positive (+)	Neoplastic cells	Cytotoxic molecule; co-expressed with CD2 in aggressive B-NHL	
CD20	Negative (−)	Neoplastic cells	Atypical; CD20-negative DLBCL; therapeutic implications	
CD138	Negative (−)	Neoplastic cells	Rules out plasma cell differentiation	
CD30	Negative (−)	Neoplastic cells	Rules out anaplastic morphology/CD30+ DLBCL	
MUM1/IRF4	Negative (−)	Neoplastic cells	Rules out post-germinal center differentiation	
ALK	Negative (−)	Neoplastic cells	Rules out ALK+ anaplastic large-cell lymphoma	
CD3	Negative (−)	Neoplastic cells	T-cell marker absent; excludes T-cell lymphoma	
CD4	Negative (−)	Neoplastic cells	Histiocytic background staining; not tumoral	
CD5	Negative (−)	Neoplastic cells	Rules out mantle cell lymphoma, CLL/SLL	
CD7	Negative (−)	Neoplastic cells	T-cell marker absent	
CD8	Negative (−)	Neoplastic cells	Cytotoxic T-cell marker absent on tumor cells	
EBV (ISH)	Negative (−)	All cells	EBER negative; viral etiology excluded	
CD103	Positive (+)	IELs	Increased IELs; reactive (celiac disease)	
TP53 (IHC)	Overexpressed	Neoplastic cells	Suggests TP53 mutation confirmed by NGS	

**Figure 3:**
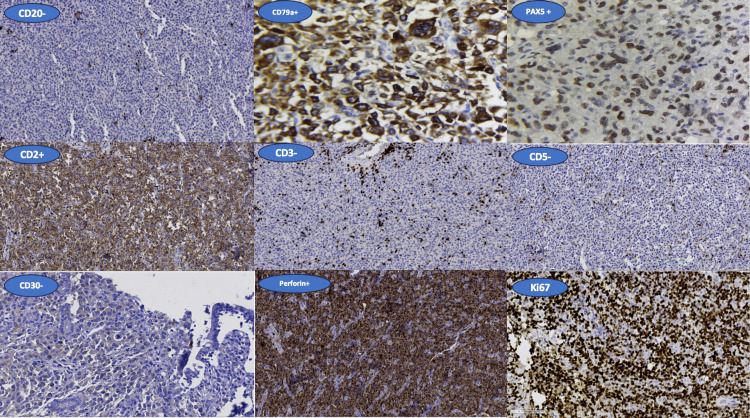
Immunohistochemical characterization of diffuse large B-cell lymphoma arising in celiac disease (x40).

Immunohistochemistry confirmed B-cell lineage through co-expression of PAX5, CD19, and CD79a, while CD20 was unexpectedly negative. Aberrant co-expression of CD2 and perforin was noted on the neoplastic cells, whereas T-cell markers CD3, CD5, and CD7 were negative. CD30, ALK, MUM1, and CD138 were all negative, excluding anaplastic large-cell lymphoma, plasmablastic lymphoma, and plasma cell neoplasms. Epstein-Barr virus was negative by Epstein-Barr virus-encoded small RNA in situ hybridization. The increased CD103+/CD8+ IELs in the residual mucosa displayed a non-aberrant phenotype, consistent with reactive expansion in active CD rather than type II refractory CD. Strong p53 overexpression on immunohistochemistry was corroborated by NGS, which identified a pathogenic TP53 mutation alongside a TET2 variant consistent with CHIP and a SETD2 germline polymorphism. IGH gene rearrangement on PCR confirmed B-cell monoclonality.

The final diagnosis was CD20-negative primary intestinal large B-cell lymphoma with TP53 mutation arising on a background of active CD. Standard R-CHOP was considered suboptimal given CD20 negativity and TP53-associated chemoresistance; CD19-targeting strategies and clinical trial enrollment were discussed at the multidisciplinary tumor board. 

## Discussion

This case illustrates a rare and diagnostically challenging primary intestinal large B-cell lymphoma arising in a young woman with chronically active CD. Several aspects make this case noteworthy, including the rarity of B-cell lymphoma in the setting of CD, the unusual immunophenotype with CD20 negativity and aberrant CD2/perforin expression, the presence of a TP53 mutation, and the coexistence of reactive intraepithelial lymphocytosis related to active CD. To the best of our knowledge, this combination of clinicopathological and molecular features has not been previously reported in the literature.

Although lymphomas associated with CD are predominantly of T-cell origin, rare cases of primary intestinal B-cell lymphomas have been reported [5,7,8]. The mechanisms underlying B-cell lymphomagenesis in CD remain incompletely understood. Chronic antigenic stimulation, persistent mucosal inflammation, cytokine dysregulation, and impaired immune surveillance are believed to contribute to malignant B-cell transformation [9,10]. Poor adherence to a gluten-free diet, as observed in our patient, likely promotes this pro-inflammatory environment and increases the risk of malignant transformation [3].

The immunophenotype in this case was particularly challenging. Despite the absence of CD20 expression, positivity for PAX5, CD19, and CD79a confirmed B-cell lineage. PAX5 is considered one of the most reliable B-cell markers and is often retained in CD20-negative lymphomas [11]. CD20-negative diffuse large B-cell lymphoma is uncommon and has important therapeutic implications, as the efficacy of rituximab depends on CD20 expression [12].

Aberrant expression of T-cell-associated markers, such as CD2 and perforin, has been reported in a small subset of aggressive B-cell lymphomas. It may lead to diagnostic confusion with T-cell or NK-cell neoplasms [12]. In the present case, negativity for CD3, CD5, and CD7 excluded a T-cell lineage and supported the diagnosis of B-cell lymphoma. However, a small subset of aggressive B-cell lymphomas may express T-cell markers, particularly CD2 and cytotoxic markers.

The TP53 mutation identified in this tumor represents an important adverse prognostic factor. TP53 alterations are associated with chemoresistance, aggressive clinical behavior, and poor survival in diffuse large B-cell lymphoma [6,13]. In our patient, the coexistence of TP53 mutation and CD20 negativity suggests a particularly high-risk biological profile and may limit the efficacy of standard rituximab, cyclophosphamide, hydroxydaunorubicin (doxorubicin), Oncovin (vincristine), and prednisone (R-CHOP) therapy [13]. Novel therapeutic approaches, including CD19-targeted therapies and CAR T-cell strategies, may represent alternatives in such cases [13,14].

The TET2 variant detected by NGS was interpreted as clonal hematopoiesis of indeterminate potential rather than a lymphoma-specific mutation. Although TET2 mutations are frequently identified in clonal hematopoiesis of indeterminate potential (CHIP), their precise impact on lymphoma biology remains uncertain [15].

Another important finding was the marked increase in CD103+/CD8+ intraepithelial lymphocytes in the surrounding mucosa. Preservation of the CD8 phenotype supported a reactive process related to active CD rather than refractory CD type II, which is characterized by aberrant intraepithelial lymphocytes and carries a high risk of progression to enteropathy-associated T-cell lymphoma [16].

Overall, this case highlights the complexity of intestinal lymphoid proliferations occurring in the setting of CD and emphasizes the importance of integrating histopathological, immunophenotypic, and molecular findings to establish an accurate diagnosis.

## Conclusions

This case highlights the diagnostic complexity of primary intestinal large B-cell lymphoma arising in the setting of poorly controlled CD. The combination of CD20 negativity, aberrant T-cell marker expression, and concurrent reactive intraepithelial lymphocytosis created substantial diagnostic challenges that required comprehensive immunohistochemical profiling, NGS, and expert pathological review. The identification of a TP53 mutation carries significant prognostic weight and argues against standard R-CHOP as an optimal therapeutic strategy, favoring individualized or trial-based approaches targeting CD19 or BCL-2 pathways. This case reinforces the importance of integrated morphological, immunophenotypic, and molecular workup in the evaluation of intestinal lymphomas and serves as a reminder that strict adherence to a gluten-free diet remains a critical and modifiable factor in reducing the risk of lymphomatous transformation in CD.
